# Cerebral oxygen saturation as outcome predictor after transfemoral transcatheter aortic valve implantation

**DOI:** 10.1007/s00392-022-02019-w

**Published:** 2022-05-04

**Authors:** Philipp C. Seppelt, Silvia Mas-Peiro, Arnaud Van Linden, Sonja Iken, Kai Zacharowski, Thomas Walther, Stephan Fichtlscherer, Mariuca Vasa-Nicotera

**Affiliations:** 1 Division of Cardiology, Department of Medicine III, University Hospital Frankfurt, Goethe University, Frankfurt am Main, Germany; 2 DZHK Partner Site Rhine-Main, German Centre for Cardiovascular Research, Berlin, Germany; 3 Department of Cardiothoracic Surgery, University Hospital Frankfurt, Goethe University, Frankfurt am Main, Germany; 4 Department of Anesthesiology, Intensive Care Medicine and Pain Therapy, University Hospital Frankfurt, Goethe University, Frankfurt am Main, Germany

**Keywords:** Valvular cardiomyopathy, Aortic stenosis, Cerebral oxygen saturation, TAVI

## Abstract

**Background:**

Cerebral oxygen saturation (ScO_2_) can be measured non-invasively by near-infrared spectroscopy (NIRS) and correlates with cerebral perfusion. We investigated cerebral saturation during transfemoral transcatheter aortic valve implantation (TAVI) and its impact on outcome.

**Methods and results:**

Cerebral oxygenation was measured continuously by NIRS in 173 analgo-sedated patients during transfemoral TAVI (female 47%, mean age 81 years) with self-expanding (39%) and balloon-expanding valves (61%). We investigated the periprocedural dynamics of cerebral oxygenation. Mean ScO_2_ at baseline without oxygen supply was 60%. During rapid ventricular pacing, ScO_2_ dropped significantly (before 64% vs. after 55%, *p* < 0.001). ScO_2_ at baseline correlated positively with baseline left-ventricular ejection fraction (0.230, *p* < 0.006) and hemoglobin (0.327, *p* < 0.001), and inversely with EuroSCORE-II ( − 0.285, *p* < 0.001) and length of in-hospital stay ( − 0.229, *p* < 0.01). Patients with ScO2 < 56% despite oxygen supply at baseline had impaired 1 year survival (log-rank test *p* < 0.01) and prolonged in-hospital stay (*p* = 0.03). Furthermore, baseline ScO_2_ was found to be a predictor for 1 year survival independent of age and sex (multivariable adjusted Cox regression, *p* = 0.020, hazard ratio (HR 0.94, 95% CI 0.90–0.99) and independent of overall perioperative risk estimated by EuroSCORE-II and hemoglobin (*p* = 0.03, HR 0.95, 95% CI 0.91–0.99).

**Conclusions:**

Low baseline ScO_2_ not responding to oxygen supply might act as a surrogate for impaired cardiopulmonary function and is associated with worse 1 year survival and prolonged in-hospital stay after transfemoral TAVI. ScO_2_ monitoring is an easy to implement diagnostic tool to screen patients at risk with a potential preserved recovery and worse outcome after TAVI.

**Graphical abstract:**

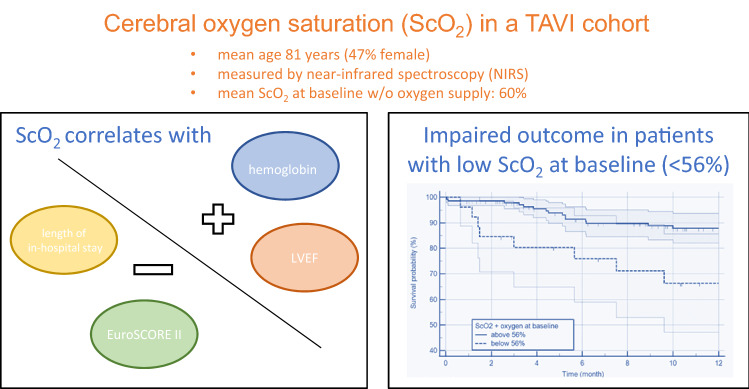

## Introduction

TAVI has become the gold standard for treatment of degenerative aortic valve stenosis in older patients with high-to-intermediate operative risks [1]. Predictors for favorable long-term survival and quality of life after TAVI are of high interest, since a relevant proportion of TAVI patients have cerebral and neurocognitive comorbidities. In a previous pilot study, we described the correlation between decline of cerebral oxygenation during rapid ventricular pacing for TAVI and postoperative delirium [2]. Real-time measured cerebral O_2_ saturation reflects cerebral perfusion and mirrors the central venous oxygen saturation, one important determinant of the systemic oxygen balance [3, 4]. Cerebral oxygen saturation can be measured non-invasively and continuously by near-infrared spectroscopy (NIRS). Methodically, NIRS measures the relative changes of the different light absorption spectra of oxygenated and deoxygenated hemoglobin. NIRS configurations used in clinical practice with sensor pads placed at the forehead determine cerebral blood saturation in a ratio of about 84% venous and 16% arterial blood [5]. One important methodological limitation of NIRS for ScO_2_ measurement is the inability to detect hypoperfusions outside of the frontal cerebral lobes. NIRS for cerebral oxygenation saturation measurement has been widely studied in different clinical settings including surgery, resuscitation and cerebral injury [6–9]. The role of cerebral oxygen saturation and its impact on outcome parameters has not yet been investigated in the setting of TAVI. In this study, we analyzed the association of intraprocedural measured cerebral oxygenation with baseline and outcome parameters in patients with aortic valve stenosis undergoing transfemoral TAVI.

## Methods

### Study design

Between July 2018 and December 2020, we measured ScO_2_ in 173 patients receiving transfemoral TAVI in analgo-sedation. All patients underwent preoperative duplex sonography of the supra-aortic vessels, echocardiography (transthoracic or transesophageal) and computed tomography for procedure planning and valve sizing. Low-flow low-gradient aortic stenosis with reduced ejection fraction was defined according to guideline recommendations: aortic valve area (AVA) < 1.0 cm2, mean transvalvular pressure < 40 mmHg, left-ventricular ejection fraction (LVEF) < 50% and stroke volume index < 35 ml/m^2^ (SVI) [10]. Carotid artery disease was defined as at least 10% stenosis according to the NASCET-Classification [11]. NASCET stenosis of > 50%, indicating moderate-to-severe carotid artery disease, were evaluated separately. Patients with symptomatic carotid artery disease or indication for revascularization were not included. Performance in activities of daily living was determined by Barthel-Index and cognitive impairment by MMSE (Mini-Mental State examination) [12]. An MMSE result below 24 points was interpreted as abnormal indicating a cognitive impairment [13]. An invasive dual-pressure analysis was performed to obtain aortic valve gradients and left-ventricular end-diastolic pressure before the first rapid ventricular pacing or valve implantation and at the end of the procedure. Postoperative delirium was assessed and diagnosed by CAM-ICU (Confusion Assessment Method for the intensive care unit) during the first 2 postoperative days or later if delirium was suspected [14]. Decision for valve intervention, selection of approach, and valve type were made by an interdisciplinary heart team consisting of cardiologists, cardiac surgeons, and anesthesiologists and further disciplines when needed. Patients in cardiogenic shock or patients requiring inotropic support prior to the procedure were excluded from analysis. Before discharge valve prothesis, function was assessed by transthoracic echocardiography. Outcome parameters were reported according to the VARC-3 criteria [15]. The study and data collection were approved by the ethics committee of the University Hospital of Frankfurt (296/16 and 19/461), and all patients gave signed and informed consent prior to intervention. Long-term follow-up information was obtained via contact with general practitioners, other hospitals, or with the patient or family directly.

### Transcatheter aortic valve implantation

TAVI procedures were performed in our hybrid operating room in Heart Team approach by an interventional cardiologist, a cardiac surgeon and a cardiac anesthesiologist. Procedures were performed exclusively under analgo-sedation using fentanyl (1–2 μg/kg body weight). One patient received remifentanil and 5 patients received midazolam additionally (intravenous 1–2 mg). Mepicavain was infiltrated at the puncture sites for local anesthesia (10–20 ml 10 mg/ml). Femoral access was obtained with re-closure devices (either Perclose ProGlide, Abbott Vascular, Abbott Park, Illinois, USA or Manta closure device, Teleflex, Pennsylvania, USA). For rapid ventricular pacing (RVP) a temporary pacing wire was placed via the femoral vein in the right ventricular apex. Retrograde passing of the aortic stenosis was performed as per interventionist standard. Before changing to a stiff pre-shaped wire for valve deployment (SAFARI^2^ Boston Scientific, Massachusetts, USA), dual-invasive pressure analysis was performed with two pigtail catheters in the aorta and left ventricle. If required, RVP was performed for pre-dilatation, for valve deployment and for prosthesis post-dilatation. Prosthesis function was evaluated by aortic angiogram and invasive dual catheter pressure analysis. At the end of the procedure, patients were transferred to an intermediate care unit and were monitored for at least 48 h post-intervention.

### Measurements of cerebral oxygenation

**Fig. 1:**
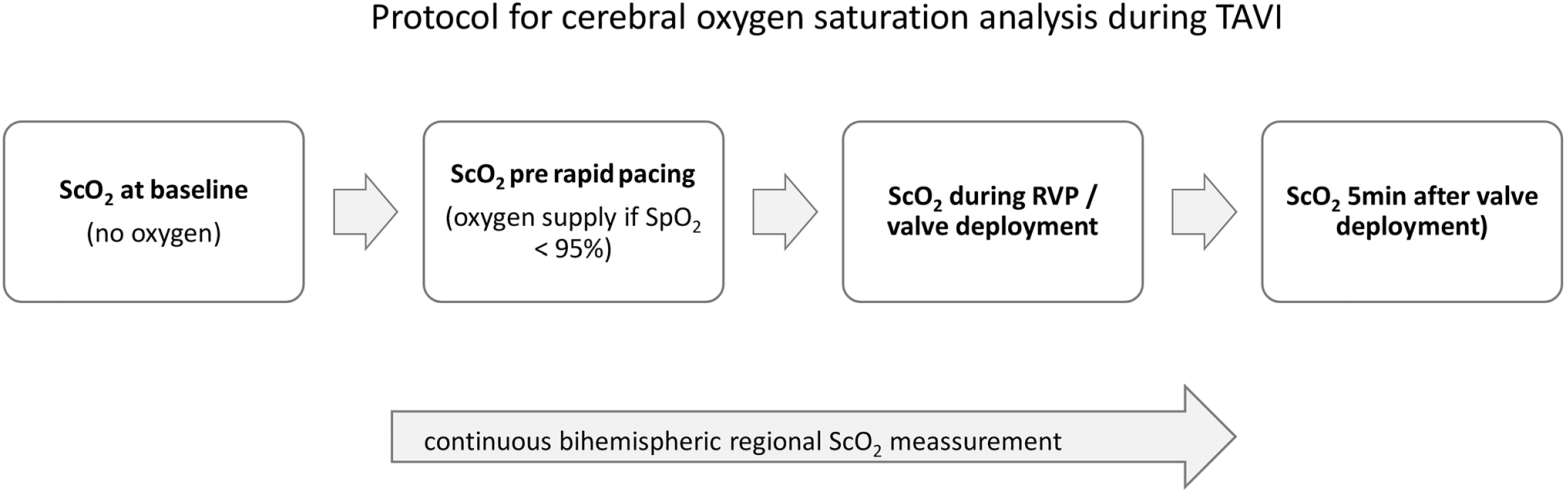
Protocol for cerebral oxygen saturation (ScO2) measurement during transfemoral TAVI procedure. *ScO*_*2*_ cerebral oxygen saturation, *SpO*_*2*_ peripheral oxygen saturation, *TAVI* transcatheter aortic valve implantation

Regional cerebral oxygenation (ScO_2_) was monitored by placing two NIRS optodes on the forehead (Root^®^, Masimo, Irvine, USA). Values for both hemispheres were documented, but the mean value was used for analysis. The baseline values were determined before induction of analgo-sedation without oxygen supply (Fig. 1). Only if the peripheral oxygen saturation (SpO_2_, measured by standard peripheral oximetry) was below 95%, patients received supplementary oxygen. The ScO_2_ values were recorded continuously and values prior, during and 5 min after last RVP or valve deployment were documented. If two or more RVPs were performed mean values were used for analysis. Additionally, the lowest and highest intraprocedural ScO_2_ were documented.

### Statistics

Continuous variables are shown as mean ± standard deviation and categorical data are shown as number (percentage). European System for Cardiac Operative Risk Evaluation Score II (EuroSCORE-II) and Barthel-Index are presented as median ± interquartile range [16]. Unadjusted differences were compared with *χ*^2^ tests for categorical variables and 2-tailed unpaired Student’s *t* tests for continuous variables. Mann–Whitney *U* Test was applied for non-parametric testing or for sample sizes < 50. Because of its robustness, non-parametric Spearman’s Rho test was used to measure the strength of association between intraprocedural ScO_2_ and baseline/outcome variables. We determined the predictive value of intraprocedural measured ScO_2_ for 1 year survival by receiver-operating characteristic curve (ROC) analysis. Perfect cut-off values were calculated using Youden index with priority on optimizing sensitivity to screen for true-positive cases (patients at risk) [17]. Long-term survival was estimated by Kaplan–Meier function and distinctions between subgroups were verified by log-rank test. Furthermore 1 year survival was analyzed by Cox proportional hazards regression model adjusted to baseline variables. In model 1, age, sex and hemoglobin at baseline, and in model 2, EuroSCORE-II and hemoglobin at baseline were included into the model as fixed variables. Model 3 contained low-flow low-gradient aortic stenosis and the type of valve prothesis as fixed variables (balloon-expandable or self-expandable valve). Intraprocedural ScO_2_ values were tested with both models en bloc in a single step (Enter mode). The a priori level of statistical significance was set at *p* < 0.05 for all analyses, which were always 2-tailed and performed with SPSS, MedCalc and R (IBM SPSS 25, Chicago, USA, MedCalc Software Ltd, Ostend, Belgium and R 3.6.1, www.r-project.org).

## Results

### Baseline characterization

The mean age in our study cohort was 81 years (47.4% female) with a median perioperative risk of 4.1% according to EuroSCORE-II (intermediate risk, Table 1). A median Barthel-Index of 90 points indicates only minor limitations in activities of daily living for most patients (19.5% of the patients < 75 points). At least mild-to-moderate carotid artery disease was present in 50 patients (28.9%) and moderate-to-severe carotid artery disease in 15 patients (8.7%). Twenty patients had a cerebral bleeding or a minor or major stroke in their medical history (11.6%). Nine patients suffered from pre-existing dementia (5.2%) and 48 patients (27.7%) had mild-to-moderate impaired cognitive function according to MMSE. Transthoracic echocardiography revealed mean baseline left-ventricular ejection fraction of 53% (Table 2). All patients underwent TAVI for severe aortic stenosis (mean aortic valve area 0.82 cm^2^) and were classified either as normal-flow high-gradient (*n* = 102, 59.3%) or as low-flow low-gradient (*n* = 32, 18.5%) aortic stenosis with a mean gradient of 43.6 mmHg and 24.5 mmHg, respectively. Anemia defined as hemoglobin (Hb) at baseline < 12 g/dl in women and < 13 g/dl in men was found in 74% in women (mean hemoglobin, Hb 11.9 g/dl) and 55% in men (mean Hb 12.4 g/dl).

**Table 1:** Patient characteristics (*n* = 173)

Female (*n*)	82	(47.4%)
Age (years)	81.0	± 6.0
Body mass index (kg/m^2^)	27.1	± 5.8
EuroSCORE-II (%)^a^	4.1	(2.2–6.8)
Barthel-Index (0–100 points)^a^	90	(80–90)
NYHA III	121	(69.9%)
NYHA IV	15	(8.7%)
Hemodialysis (*n*)	5	(2.9%)
Previous cardiac decompensation (*n*)	79	(45.7%)
Diabetes mellitus (*n*)	62	(35.8%)
Atrial fibrillation (*n*)	58	(33.5%)
Permanent pacemaker (prior to TAVI, *n*)	18	(10.4%)
Coronary heart disease (*n*)	102	(59%)
Previous PCI (*n*)	70	(40.7%)
Previous myocardial infarction (*n*)	33	(19.1%)
Cerebral arterial disease (*n*)	50	(28.9%)
Mild-to-moderate	35	(20.2%)
Moderate-to-severe	15	(8.7%)
Stroke (*n*, minor or major)	20	(11.6%)
Dementia (*n*)	9	(5.2%)
Cognitive impairment (MMSE < 24 points, *n*)	48	(27.7%)
Peripheral artery disease (*n*)	59	(34.1%)
Chronic lung disease (*n*)	47	(27.1%)
Hemoglobin (g/dl)	12.1	± 1.9
Creatinine (mg/dl)	1.29	± 0.9
NT-proBNP (ng/l)	4776	± 13,021
High-sensitive Troponin-T (ng/l)	52	± 110

Data shown as *n* (percentage) or mean (± standard deviation)

*LVEF* left-ventricular ejection function, *PCI* percutaneous coronary intervention

^a^EuroSCORE-II and Barthel-Index are presented as median (interquartile range)

**Table 2:** Baseline echocardiography

LVEF (%)	53.0	± 12.0
Aortic valve area (cm^2^)	0.82	± 0.27
Mean aortic valve gradient (mmHg)	40	± 16
Maximum aortic valve gradient (mmHg)	62	± 24
Severe aortic valve insufficiency (*n*)	9	(5.3%)
Severe mitral valve insufficiency (*n*)	20	(11.6%)
Severe tricuspid valve insufficiency (*n*)	14	(8.1%)
Low-flow low-gradient aortic valve stenosis	32	(18.5%)
TAPSE (mm)	21	± 6
Systolic pulmonary artery pressure (mmHg)	41	± 14

Data shown as *n* (percentage) or mean (± standard deviation)

*LVEF* left-ventricular ejection function, *TAPSE* tricuspid annular plane systolic excursion

### TAVI procedure

In 105 patients (60.7%) a self-expandable and in 68 patients (39.3%) a balloon-expandable prothesis was implanted (Table 3). In 29 implantations, no RVP (16.8%) and in 98 implantations one RVP was conducted (56.6%; two or more RVP, in *n* = 46, 26.6%).

**Table 3:** Procedural outcome (*n* = 173) according to VARC-3 [15]

Aortic valve prostheses		
Edwards S3/S3 Ultra (*n*)	68	(39.3%)
Boston scientific ACURATE neo (*n*)	59	(34.1%)
St. Jude Portico (*n*)	23	(13.3%)
Medtronic evolute Pro/R (*n*)	23	(13.3%)
Valve in valve procedure	4	(2.3%)
Valve size (mm)	26.63	± 2.6
RVP (*n* of the patients) *n* = 0	29	(16.8%)
RVP (*n* of the patients) *n* = 1	98	(56.6%)
RVP (*n* of the patients) *n* = 2	41	(23.7%)
RVP (*n* of the patients) *n* = 3	4	(2.3%)
RVP (*n* of the patients) *n* = 4	1	(0.6%)
Balloon pre-dilatation (*n* of the cases)	91	(52.6%)
Balloon post-dilatation (*n* of the cases)	35	(52.6%)
Contrast medium (ml)	85.7	± 49.0
Fluoroscopy time (min)	13.2	± 9.0
Postoperative delirium (*n*)	45	(26.0%)
Serious access site vascular complication (*n*)	5	(2.9%)
Severe prosthetic aortic valve regurgitation (*n*)	1	(0.6%)
Mean aortic valve gradient (mmHg)	9.5	± 6.2
Maximum aortic valve gradient (mmHg)	17.3	± 9.5
Stroke (*n*)	4	(2.3%)
Valve reoperation (*n*)	4	(2.3%)
Need for new pacemaker (*n*)	33	(19.1%)
30 day mortality (*n*)	3	(1.7%)
Days on Intensive Care Unit (days)	3.3	± 2.3
Days in hospital (days)	8.9	± 7.2

Data shown as *n* (percentage) or mean ± standard deviation

*RVP* rapid ventricular pacing

### Intraprocedural measurement of regional cerebral oxygenation by NIRS

**Fig. 2:**
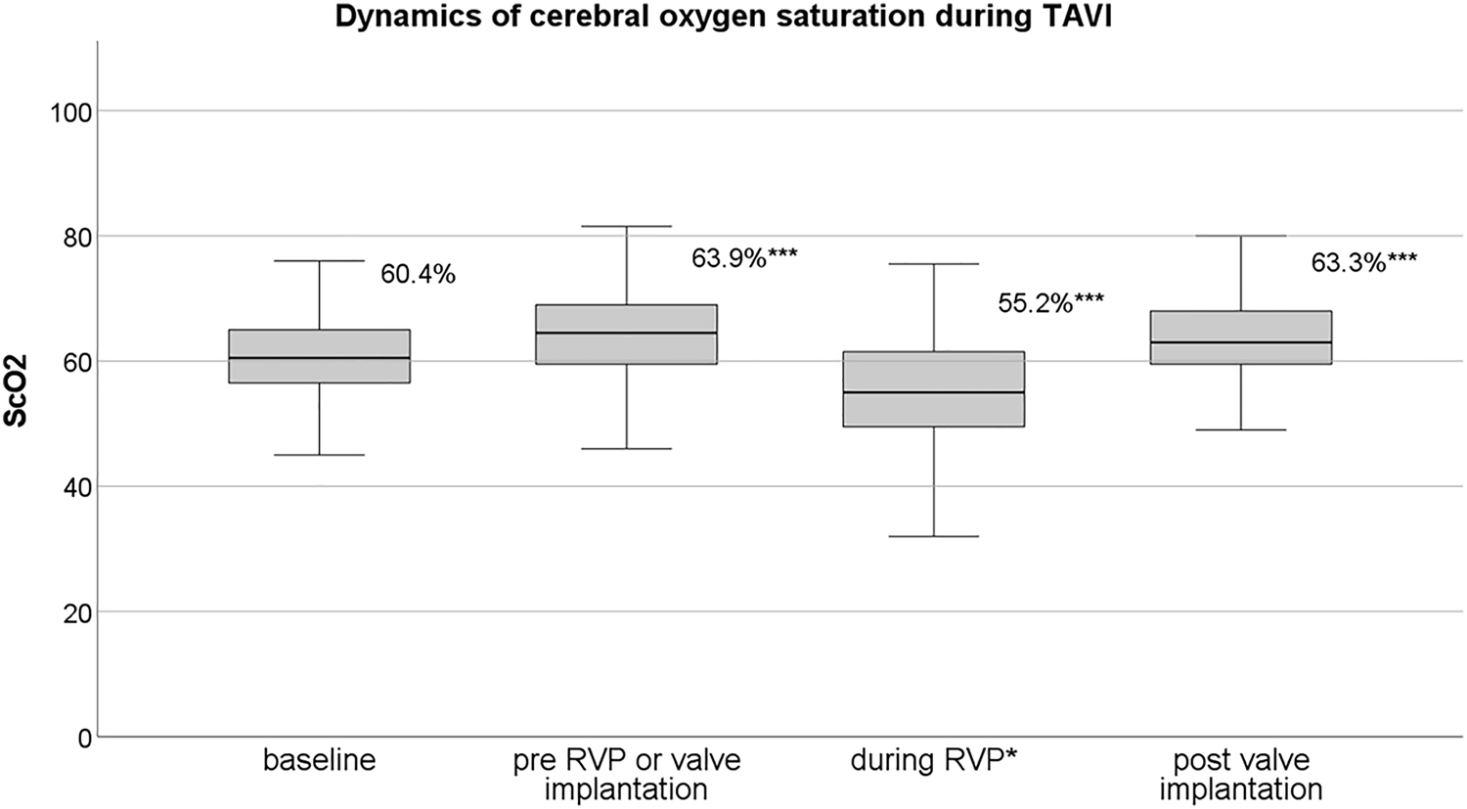
Course of cerebral oxygen saturations during transfemoral TAVI. Mean ScO_2_ values measured at both frontal hemispheres at the different time points during TAVI procedure. *ScO*_*2*_ cerebral oxygen saturation, *SpO*_*2*_ peripheral oxygen saturation, *RVP* rapid ventricular pacing, *TAVI* transcatheter aortic valve implantation. *During RVP or valve deployment; ***p < 0.001, Student’s *t *test, in comparison to the previous timepoint

Mean baseline ScO_2_ was 60.4% and increased after oxygen supply (63.9%, *p* < 0.001; Fig. 2), but did not differ between cerebral hemispheres (left 60.3% vs. 60.5% right, *p* = 0.745). All patients were analgo-sedated, spontaneously breathing and 95.4% of the patients received oxygen supply (mean 5.3 l/min, aimed peripheral oxygen saturation > 95%). During RVP for balloon dilatation or valve implantation, ScO_2_ declined significantly (63.9% vs. 55.2%, *p* < 0.001) and raised to normalized values 5 min after RVP or valve implantation (55.2% vs. 63.6%, *p* < 0.001). In cases with at least one RPV, baseline ScO_2_ did not differ, but ScO_2_ nadir was lower compared to cases without RVP (52.0% vs. 55.1%, *p* = 0.03).

### Correlation of cerebral oxygen saturation with baseline characteristics

Baseline ScO_2_ correlated inversely with estimated perioperative risk (EuroSCORE-II, − 0.285, *p* < 0.001) and with the cardiac biomarkers NT-proBNP (N-terminal pro-brain natriuretic peptide, − 0.334, *p* < 0.001) and high-sensitive Troponin-T ( − 0.256, *p* < 0.01). Furthermore, Hb concentration correlated significantly with baseline ScO_2_ (0.327, *p* < 0.001) and patient with baseline ScO_2_ below the median (< 60.5%) had significant lower Hb concentration (11.6 vs. 12.6 g/dl, *p* < 0.001). LVEF was found to correlate with ScO_2_ at baseline (0.230, *p* < 0.01), lowest measured ScO_2_ (0.312, *p* < 0.001) and delta highest-lowest ScO_2_ ( − 0.359, *p* < 0.001). Patients with low-flow low-gradient aortic valve stenosis had lower ScO_2_ at baseline without (57.0 vs. 61.2%, *p* < 0.001) and with oxygen supply (60.8 vs. 64.7%, *p* < 0.01), during RVP (51.2 vs. 56.1%, *p* < 0.01) and lowest documented ScO_2_ (49 vs. 53.6%, *p* < 0.01). In the entire cohort, lower baseline and lowest intraprocedural ScO_2_ were inversely associated with longer in-hospital stay ( − 0.229, *p* < 0.01 and  − 0.185, *p* = 0.015 respectively) and delta highest–lowest ScO_2_ correlated with the development of postoperative delirium (0.286, *p* < 0.001). If a decline in ScO_2_ of > 20% occurred during the procedure, patients suffered more often from postoperative delirium (34.0% vs. 15.8%, *p* < 0.01). Patients with baseline ScO_2_ below 56% stayed significantly longer in hospital (14.9 vs. 8.0 days, *p* = 0.030). However, we found no association of ScO_2_ with age, Barthel-Index, dementia or cognitive impairment. Furthermore, ScO_2_ at baseline did not differ in patients with mild-to-moderate (60.4%) or moderate-to-severe (62%) compared to patients with no carotid artery disease (60.5%, *p* = 0.466 and *p* = 0.360, respectively). However, we observed a weak negative correlation of carotid artery disease with post-rapid pacing ScO2 (correlation efficient  − 0.170, *p* = 0.024).

### Survival analysis

**Fig. 3:**
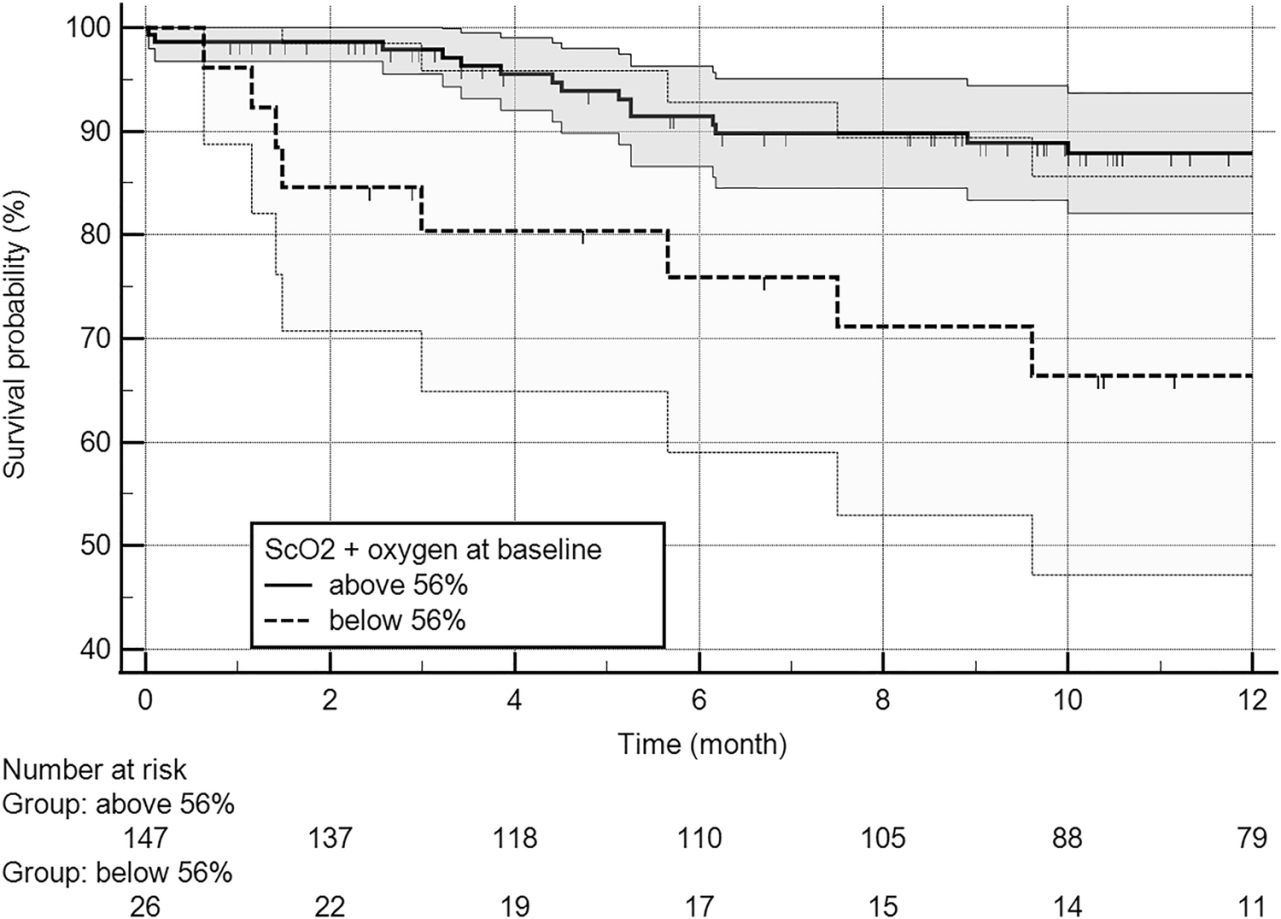
Kaplan–Meier survival analysis Estimated survival generated by Kaplan–Meier survival analysis comparing patients with ScO_2_ at baseline with oxygen supply < 56% and > 56%. Survival distributions of both groups were compared by log-rank test after 1 year (*p* < 0.01)

Mean follow-up was 400 days, and cumulative 1 year survival 82.9%. Completeness of follow-up was 100% after 30 days and 89.6% after 1 year. There was a tendency for an impaired survival of patients with low-flow low-gradient aortic stenosis after 1 year (log-rank test, *p* = 0.09) and a significantly reduced survival over the complete follow-up (*p* = 0.023). ROC analysis revealed baseline ScO_2_ (with oxygen supply) as a predictor for 1 year survival (AUC 0.66, *p* < 0.01) and ScO_2_ of 56% was determined as optimized cut-off value to screen for patients with favorable 1 year survival (calculated by Youden Index, sensitivity 0.91/specificity 0.41). Survival analysis revealed a significantly reduced 1 year survival (87.9% vs. 66.4%, log-rank test *p* < 0.01; Fig. 3)  and overall survival for patients with ScO_2_ at baseline < 56% with oxygen supply (*p* = 0.013). These patients had higher EuroSCORE-II (3.9 vs. 7.3%, *p* < 0.001), but did not differ in age (80.9 vs. 81.3 years, *p* = 0.636). An intraprocedural ScO_2_ drop by more than 20% of the highest measured ScO_2_ value was not associated with worse 1 year survival (81.5% vs. 82%, log-rank test *p* = 0.901) or prolonged in-hospital stay (median 7.0 and 7.0 days, *p* = 0.329). Instead, ScO_2_ at baseline under oxygen supply was found to be a predictor for 1 year survival independent of age and sex (multivariable adjusted Cox regression, *p* = 0.020, hazard ration, HR 0.94, 95% CI 0.90–0.99) and independent of overall perioperative risk estimated by EuroSCORE-II and hemoglobin (*p* = 0.03, HR 0.95, 95% CI 0.91–0.99, Table 4). Intervention with balloon-expandable valve was a predictor for improved 1 year survival but estimated 1 year survival was not significantly better (log-rank test, *p* = 0.144, 80.6% vs 89.2%) and patients were younger (79.8 vs. 82.1, *p* = 0 < 0.01).

**Table 4:**
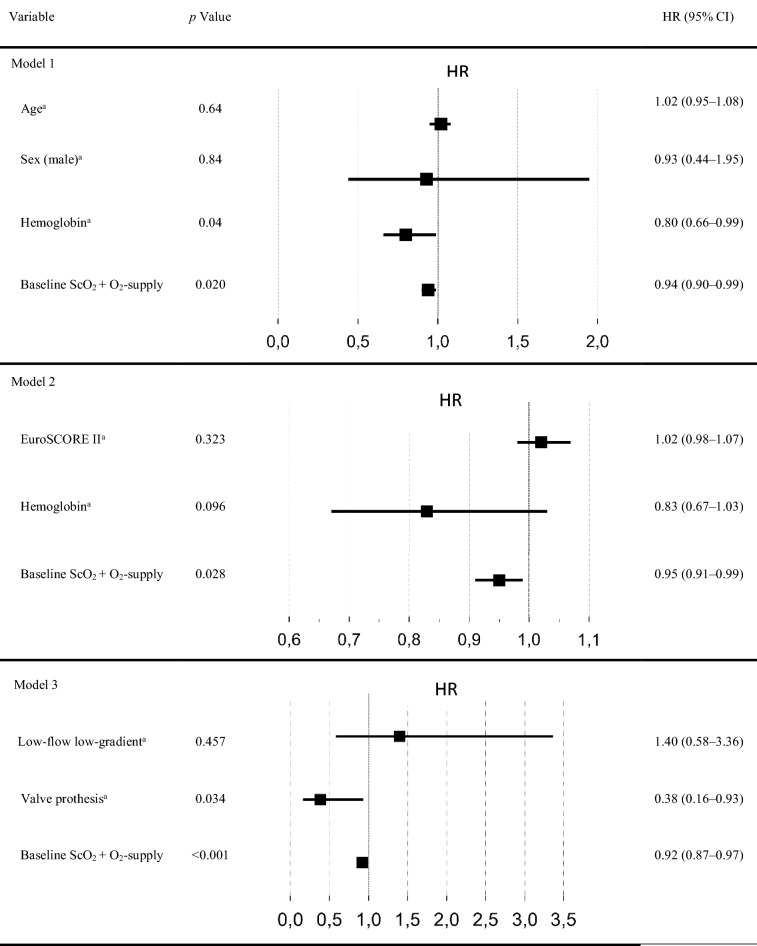
Cox regression for 1 year survival analysis

Multivariable Cox regression for 1 year survival analysis

ScO2 regional cerebral oxygen saturation, CI confidence interval, HR hazard ratio

^a^ Variables were fixed in the models for baseline adjustment. ScO2 at baseline with oxygen supply shows an influence on long-term survival independent of age and sex (model 1) and independent of perioperative risks (estimated by EuroSCORE II) and hemoglobin (model 2). Valve prothesis, in favor for balloon-expandable valves, is a predictor for 1 year survival (model 3)

### Discussion

There is no sufficient experience with how to interpret cerebral oxygen saturation during TAVI procedure and how it can be used to improve risk stratification and outcome prognosis. We identified correlations between pre-existing risk factors mirroring cardiovascular functionality and ScO_2_ at baseline. Furthermore, patients with oxygen supply and ScO_2_ < 56% at baseline had worse 1 year survival. To the best of our knowledge, this is the first study to report cerebral oxygen saturation during transfemoral TAVI systematically in a larger cohort.

With our study, we aimed to address two questions. First, does ScO_2_ correlate with pre-existing risk factors in an all-comer TAVI cohort? Second, is ScO_2_ a valuable and independent predictor for long-term survival after TAVI?

We found a significant correlation of ScO_2_ at baseline with markers for cardiovascular diseases. High-sensitive Troponin-T, NT-proBNP and EuroSCORE-II, a valid risk score estimating the 30 day mortality after cardiac surgery, correlated inversely and left-ventricular ejection function, serum hemoglobin concentration correlated positively with baseline ScO_2_. In-depth insights about cerebral oxygen saturation and cardiovascular functionality have been described in the setting of cardiac arrest and low cardiac output. Coherent to our result, a study by Skhirtladze et al. reported an association of compromised left-ventricular pump function and diminished ScO_2_ during threshold testing with concomitant induction of cardiac arrest in patients undergoing elective implantation of a cardioverter/defibrillator, a similar clinical setting to RVP or valve deployment during TAVI [18]. In detail, patients with LVEF < 30% exhibited the lowest ScO_2_ values and had the highest incidence of critical cerebral desaturations, defined by the authors as > 20% drop from baseline or ScO_2_ values < 50%.

A prospective study conducted by Robu et al. measured ScO_2_ at baseline in 1616 patient undergoing cardiac interventions [19]. Baseline ScO_2_ was observed to decrease with advanced age and was lower in women. Moreover, hemoglobin showed a significant association with ScO_2_. Blood loss during surgery is known to reduce cerebral oxygen saturation; moreover, the correlation of serum hemoglobin concentration and ScO_2_ is linear [6, 20, 21]. We also examined a strong correlation between hemoglobin and ScO_2_. Furthermore, our mean ScO_2_ at baseline was nearly identical to the baseline value reported by Robu et al. (60.0% in patients > 75 years vs. 60.4% in our cohort) [19]. One of the earlier and grand designed studies investigating ScO_2_ and its correlation to baseline parameters was conducted by Heringlake et al. in 1178 patients scheduled for on-pump cardiac bypass surgery [22]. In line with our results, the authors found an inverse correlation of high-sensitive Troponin-T, NT-proBNP and EuroSCORE-II with ScO_2_ at baseline. Since high-sensitive Troponin-T is not only a marker of myocardial injury, but comparable to NT-proBNP a measure of global cardiovascular dysfunction, ScO_2_ is a valid parameter mirroring cardiovascular dysfunction in general [23, 24]. In an early invasive study by Paquet et al., mean baseline ScO_2_ was the superior predictor for left-ventricular systolic dysfunction evaluated by transesophageal echocardiography compared to hemodynamic variables determined by pulmonary artery catheterization [25].

In addition, ScO_2_ is known to correlate with mixed venous oxygen saturation in different clinical settings, an accepted surrogate parameter for the ratio between oxygen delivery and demand [3, 4]. This suggests that ScO_2_ not only reflects the cerebral, but also the systemic oxygen balance. In summary, impaired cardiovascular and cardiopulmonary function is reflected by cerebral oxygen saturation.

Besides correlation of ScO_2_ with baseline parameters for cardiovascular functionality, we describe impaired 1 year survival in patients with oxygen supply and ScO_2_ < 56% at baseline. Moreover, ScO_2_ at baseline under oxygen supply is a predictor for 1 year survival independent of age and sex and independent of overall perioperative risks estimated by EuroSCORE-II and hemoglobin. The predictive value of ScO_2_ for survival has been described in different clinical settings.

A longitudinal study in patients with coronary artery disease demonstrated that a decline of ScO_2_ during exercise corresponds with future adverse cardiac events and cardiac death throughout an observational period of 3–4 years [26]. In patients with cardiac arrest, higher ScO_2_ levels at initiation of cardiopulmonary resuscitation (CPR) and during CPR were positive predictors for survival and reflect high-quality CPR [27]. A multicenter prospective study that included 504 out-of-hospital cardiac arrest victims who were still undergoing CPR on hospital arrival reported an association of higher cerebral oxygen saturations with return of spontaneous circulation and a perfect cut-off point for neurologically favorable survival to hospital discharge with ScO_2_ > 50% under CPR [9].

In 2011, Heringlake et al. presented evidence that baseline cerebral oxygen saturation is an independent risk factor for 30 day and 1 year mortality in patients undergoing on-pump cardiac surgery [22]. But more important, failure of oxygen supplementation to increase ScO_2_ beyond a cut-off value of 50% was a strong predictor for higher 30 day morbidity and mortality. In line with our cut-off value for 1 year survival at 56%, this emphasizes the potent diagnostic value of baseline ScO_2_ monitoring to screen for non-oxygen responders and patient at risk for impaired mid- and long-term survival.

The concept of monitoring the brain as an index organ in patients with cardiovascular diseases is not novel [28]. Besides its value as prognostic marker, it remains an open question, whether monitoring or even optimizing ScO_2_ during TAVI might improve the postoperative outcome and survival. So far, the best insights derived from randomized controlled trials in the setting of cardiac surgery with target therapies to optimize cerebral oxygen saturation. However, the results are conflicting: the results of a meta-analysis from 2017 did not support the hypotheses that cerebral NIRS-based algorithms have clinical benefits in cardiac surgery [29]; in contrast, a recent randomized controlled trial reported better memory outcome in the target therapy group but failing to improve morbidity and mortality endpoints [7]. In our study, a ScO_2_ decline > 20% during the procedure was not associated with worse 1 year survival or prolonged in-hospital stay. Especially, patients with low ScO_2_ at baseline not responding to oxygen supply have an impaired long-term outcome. In general, symptomatic severe aortic stenosis has a dismal prognosis, and valve intervention improves survival and quality of life significantly [30, 31]. Current guidelines strongly recommend early intervention in all patients [10, 32]. Therefore, omitting the intervention in case of low and non-responding ScO_2_ at baseline is not justifiable. ScO_2_ monitoring is a non-invasive and easy to implement diagnostic tool for preoperative risk assessment in patients scheduled for TAVI. We hypothesize that optimization of pretreatment is the key to improve results after TAVI.

Low ScO_2_ at baseline can be addressed by several strategies, e.g., by avoiding perioperative anemia and oxygen deficit. Furthermore, pretreatment of heart failure should be optimized and elective TAVI procedure should not be performed during acute decompensation and congestion. With this recent study, we could confirm the results of our previous pilot study, that patients with intraprocedural ScO_2_ decline of > 20% suffered more often from postoperative delirium [2]. For patients at high risk of developing a postoperative delirium, long and repetitive RVP as well as repetitive re-sheathing of a self-expandable valve should be avoided to reduce an intraprocedural ScO_2_ drop. Besides anatomical and morphological characteristics, the risk for intraprocedural cerebral desaturation should be considered to select the optimal valve system for each individual patient.

Our study has important limitations. We carried out a prospective single-center study with a heterogenous all-comer patient cohort. Although we have institutional ratified protocols for analgo-sedation during TAVI, individual sedation level is not comparable, but has an impact on cerebral oxygenation itself. Our results cannot be interpreted in the setting of anesthetized patients. Furthermore, ScO_2_ values measured in this study were generated by the Masimo oximeter and therefore not transferable to other oximetry systems.

## Conclusion

Non-invasively measured cerebral oxygen saturation mirrors cardiovascular functionality. During TAVI, a baseline ScO_2_ < 56% with oxygen supply is associated with reduced 1 year survival and ScO_2_ correlates inversely with prolonged in-hospital stay. Monitoring cerebral oxygen saturation by near-infrared spectroscopy is an easy diagnostic tool for screening patients with impaired outcome after TAVI.

## Abbreviations

CAM-ICU: Confusion Assessment Method for the ICU
EuroSCORE-II: European System for Cardiac Operative Risk Evaluation Score II
Hb: Serum hemoglobin concentration
LVEF: Left-ventricular ejection fraction
MMSE: Mini-Mental State examination
NIRS: Near-infrared spectroscopy
NT-proBNP: N-terminal pro-brain natriuretic peptide
RVP: Rapid ventricular pacing
ScO_2_: Cerebral oxygen saturation
SpO_2_: Peripheral oxygen saturation
SVI: Stroke volume index–stroke volume/body surface area
TAVI: Transcatheter aortic valve implantation
